# Identification and Characterization of* Escherichia coli*,* Salmonella *Spp.,* Clostridium perfringens*, and* C. difficile* Isolates from Reptiles in Brazil

**DOI:** 10.1155/2019/9530732

**Published:** 2019-05-27

**Authors:** Carolina Pantuzza Ramos, Jordana Almeida Santana, Fernanda Morcatti Coura, Rafael Gariglio Clark Xavier, Carlos Augusto Gomes Leal, Carlos Augusto Oliveira Junior, Marcos Bryan Heinemann, Andrey Pereira Lage, Francisco Carlos Faria Lobato, Rodrigo Otávio Silveira Silva

**Affiliations:** ^1^Departamento de Medicina Veterinária Preventiva, Escola de Veterinária, Universidade Federal de Minas Gerais, 31270-901 Belo Horizonte, Brazil; ^2^Departamento de Ciências Agrárias, Instituto Federal de Minas Gerais, 38900-000 Bambuí, Brazil; ^3^Departamento de Medicina Veterinária Preventiva e Saúde Animal, Faculdade de Medicina Veterinária e Zootecnia, Universidade de São Paulo, 05508-270 São Paulo, Brazil

## Abstract

Considering the increasing popularity of reptiles as pets and their possible role as reservoirs of pathogenic microorganisms, the aim of this study was to isolate* Escherichia coli, Salmonella *spp*., Clostridium perfringens*, and* C. difficile *strains from reptiles in Brazil and to characterize the isolated strains. The characterization was based on phylogenetic typing of* E. coli*, identification of virulence genes of* E. coli, C. perfringens, *and* C. difficile*, serotyping of* Salmonella *spp., ribotyping and MLST of* C. difficile *and antimicrobial susceptibility test of pathogenic strains. Cloacal swabs were collected from 76 reptiles, of which 15 were lizards, 16 chelonians, and 45 snakes, either living in captivity, in the wild, or as companion animals.* E. coli *was isolated from 52 (68.4%) reptiles, of which 46 (88.4%) were characterized as phylogroup B1. The virulence factor CNF1 of* E. coli* was found in seven (9.2%) sampled animals, whereas the gene of EAST1 was found in isolates from two (2.6%) reptiles. Three isolates positive for CNF1 were resistant to cephalothin, one of which was also resistant to ciprofloxacin, trimethoprim/sulfamethoxazole, and chloramphenicol, being then classified as multidrug resistant strain (MDR).* Salmonella enterica* was identified in 26 (34.2%) reptiles, of which 13 belonged to the subspecies* enterica. *Serotypes such as* S. *Mbandaka,* S. *Panama,* S*. Infantis,* S. *Heidelberg, and* S. *Anatum were identified. One isolate of* S. enterica *subsp.* houtenae* was resistant to cephalothin and ciprofloxacin.* C. perfringens *type A was isolated from six (7.8%) animals.* C. difficile *was isolated from three (3.9%) reptiles. Two of these isolates were toxigenic and classified into ribotypes/MLST 081/ST9 and 106/ST42, which have been previously reported to infect humans. In conclusion, reptiles in Brazil can harbor toxigenic* C. difficile* and potentially pathogenic* E. coli* and* Salmonella enterica* subsp.* enterica*, thus representing a risk to human and animal health.

## 1. Introduction

Several countries have shown an increase in reptiles as companion animals over the years. From 2001 to 2016, the number of households in the United States (US) with reptiles, such as turtles, snakes, and lizards, as pets increased from 1.7 to approximately 4.7 million [1]. Today, almost 4% of US homes have reptiles as pets [1, 2]. This same trend can be also seen in European countries, with a growing population of pet reptiles [3, 4]. Based on data from the latest census of companion animal population in Brazil, it was ranked as the country with the 9^th^ largest number of domesticated reptiles, with approximately 2.2 million animals [5].

The close contact between reptiles and humans is a public health concern since these animals have been characterized as carriers of zoonotic agents, mainly* Salmonella *spp., which is associated with human salmonellosis [2]. In fact, reptiles are responsible for approximately 6% of sporadic cases of human salmonellosis [6]. In addition to* Salmonella *spp., other zoonotic bacteria of the genera* Mycobacterium*,* Chlamydia,* and* Leptospira* have been associated with reptiles [7]. Some reports have also shown* Escherichia coli *carriage by reptiles and one study described the phylogenetic group B1 as more common in these animals [8, 9]. Curiously, studies have shown B1 phylogroup commonly associated with diarrheogenic pathovar, although there is no study focusing on detection of pathogenic* E. coli *in reptiles' isolates [10].* C. difficile *is an emerging pathogen responsible for the majority of nosocomial diarrhea cases in humans [11]. Interestingly, a strain of* C. difficile *was recently isolated from a lizard (*Pogona vitticeps*), being the first report of this agent in reptiles, although no toxigenic potential was found in this isolate [12]. Also, enterotoxigenic* C. perfringens, *responsible for human disease, was already found in a tortoise with diarrhea, but potential risk of reptiles is cloudy by the absence of studies with* C. perfringens* strains isolated from these animals [13].

Despite the increasing popularity of reptiles as pets, in Brazil, little is known about their carriage of potentially pathogenic microorganisms, including the main subspecies and serotypes of* Salmonella *spp. Thus far, there have been few studies focusing on detecting important human and animal enteropathogens in reptiles. Therefore, the present study aimed to realize an investigation into the carriage of* E. coli, Salmonella *spp.,* C. perfringens*, and* C. difficile *in fecal material of reptiles living in different habitats in Brazil and to characterize the isolates.

## 2. Material and Methods

### 2.1. Samples

Fecal material was obtained from swabs of cloacae from reptiles between July 2016 and September 2017. A convenience sampling of 76 apparently healthy reptiles after clinical examination was performed, consisting of samples obtained from 60 scaled reptiles (order Squamata), comprising 15 lizards (suborder Sauria) and 45 snakes (suborder Serpentes), and 16 chelonians (order Chelonii)—ten from the suborder Pleurodira and six from the suborder Cryptodira. The reptiles were selected from the following habitats: private owners volunteers (*n *= 23), free-living from metropolitan area of Belo Horizonte captured for monitoring (*n *= 37), and captivity (*n* = 16) randomly selected from the Wildlife Screening Center (CETAS) and Ezequiel Dias Foundation (FUNED). For sampling procedures, a sterile swab (BactiSwab; Remel, Lenexa, KA, USA) was introduced 5–6 cm into the cloaca and rotated five times, as described by Ives et al. [14]. The swab was vigorously agitated in 500 *μ*L of phosphate-buffered saline (PBS), stored in a transport box with ice packs and transported to the Bacterial and Research Laboratory of Veterinary School of Federal University of Minas Gerais for immediate processing. The study was approved by the Ethical Committee on Animal Use of UFMG (CEUA) under the protocol 238/2015 and by Instituto Chico Mendes de Conservação da Biodiversidade (ICMBio) under the protocol 49195-1.

### 2.2. Escherichia coli

For* E. coli* isolation, samples were plated onto MacConkey agar (Difco, USA) and incubated for 24 h at 37°C and characteristic lactose-fermenting colonies were identified using the EPM-MILI-Simmons Citrate Enterobacteriaceae identification test [15]. To identify the phylogenetic groups of* E. coli *(A, B1, B2, C, D, E, or F), a quadruplex PCR commonly used to characterize* E. coli *was performed [16]. Characterization of pathovars such as enterotoxigenic* E. coli *(ETEC), Enteropathogenic* E. coli* (EPEC), Enterohemorrhagic* E. coli *(EHEC), Shiga-toxin producing* E. coli *(STEC), Necrotoxigenic* E. coli *(NTEC), Enteroinvasive* E. coli *(EIEC), Diffusely Adherent* E. coli *(DAEC), and Enteroaggregative* E. coli *(EAEC) was also performed according to the presence of specific virulence genes. Genes encoding virulence factors associated with each* E. coli *pathovars was identified by PCR. The reference strains used were EDL933 (*eae*^*+*^,* stx1*^*+*^,* stx2*^*+*^,* hlyA*^*+*^,* iha*^*+*^,* toxB*^*+*^,* efa1*^*+*^), EAEC O42 (*ast1*^*+*^,* aggR*^*+*^,* aaf*^*+*^,* pet*^*+*^), B41 (*f41*^*+*^*, f5*^*+*^,* sta*^*+*^), S5 (*f17*^*+*^*, cnf2*^*+*^), NTEC-1 (*cnf1*), STECLBA05 (*saa*^*+*^), E2348/69 (*bfpA*^*+*^), PA58 (*aidaI*^*+*^), EIEC (*ipaH*^*+*^), 2568 (*stb*^*+*^*, f18*^*+*^*, stx2e*^*+*^), 2569 (*lt*^*+*^*, k88*^*+*^), 2570 (*987p*^*+*^), ECSTh (*sth*^*+*^*), *4833 (*cs2*^*+*^*, cs3*^*+*^), PB176 (*cs1*^*+*^), E17018A (*cs5*^*+*^*, cs6*^*+*^), H10407 (*cfa1*^*+*^), E8775 (*cs4*^*+*^) and 29 (*cs12*^*+*^). The primers used to detect virulence genes associated with diarrheagenic* E. coli *were described in Table 1S of Supplementary Material.

### 2.3. Salmonella Spp.

For isolation of* Salmonella* spp., samples were plated onto Hektoen enteric agar (Oxoid, USA) and XLT4 agar (Prodimol Biotechnology, Brazil) and incubated at 37°C for 18 - 24 h. Additionally, samples were also preenriched in tetrathionate broth (Oxoid, USA) at 37°C for 24 h prior to plating on Hektoen enteric agar (Difco, USA). Sulfite-reducing colonies of* Salmonella *spp. were identified by genus-specific PCR using the reference strain ATCC 14028 [17]. Strains confirmed as* Salmonella *spp. were differentiated into species and subspecies [18] and afterwards serotyped by antigenic characterization based on the White-Kauffmann-Le Minor scheme at the Brazilian National Reference Laboratory of Enterobacteria of Oswaldo Cruz Foundation (FIOCRUZ), Brazilian Ministry of Health, being serotyping the most common method to differentiate strains of* Salmonella *[19]. The antigenic characterization was performed by slide agglutination with somatic (O), flagellar (H), and occasionally capsular (Vi) poly- and monovalent antisera. The identification of specific serovars was performed and represented according to the criteria reported by Grimont and Weill [20]. After identification, the antibiotic resistance patterns of pathogenic* E. coli *and* Salmonella *subspecies or serotypes were evaluated by the disc diffusion method according to the Clinical and Laboratory Standards Institute (CLSI) manual VET01-A4 [21]. Briefly, four to five isolated bacterial colonies were incubated in Mueller-Hinton broth (Difco, USA) at 35°C until obtaining turbidity comparable to that of the 0.5 McFarland standard. The suspension was inoculated over the surface of a Mueller-Hinton agar plate, followed by the application of the drug-impregnated disks. The plates were incubated at 35°C ± 2°C for 16 to 18 hours and the size of inhibition zones was interpreted as recommended by the VET01-A4 [21]. The antibiotic disks used were ciprofloxacin (5 *μ*g), trimethoprim/sulfamethoxazole (25 *μ*g), chloramphenicol (30 *μ*g), ceftriaxone (30 *μ*g), and cephalothin (30 *μ*g) (DME, Brazil), and the control used for antimicrobial susceptibility was* E. coli *ATCC 25922.

### 2.4. Clostridium perfringens

The isolation of* C. perfringens* was performed according to Silva et al. [22]. Briefly, each sample was inoculated into 10 mL of brain heart infusion broth (Difco, USA) for enrichment. After incubation at 37°C for 24 h in an anaerobic atmosphere, 10 *μ*L of the culture was plated onto SPS agar (Difco, USA) and anaerobically incubated at 35 ± 2°C for 24 - 48 h. After isolation, at least three rounded sulfite-reducing colonies were subjected to PCR for the detection of genes encoding* C. perfringens *major toxins alpha (*cpa*), beta (*cpb*), epsilon (*etx*), iota (*ia*), additionally to beta-2 toxin (*cpb-2*), and enterotoxin (*cpe*), associated with human and animals' disease [23]. Also, PCR protocols for detection of additional virulence factors, such as NetB- and NetE-, NetF- and NetG-encoding genes were used, as described in Table 1S of Supplementary Material. The following* C. perfringens* strains were used as controls: BAA1481 (*cpa*^+^,* ia*^+^), ATCC 3626 (*cpa*^+^,* cpb*^+^,* etx*^+^,* cpb2*^+^, and* pfoA*^+^), D7 (*cpe*^+^,* netE*^+^,* netF*^+^,* netG*^+^), and CP149 (*netB*^+^).

### 2.5. Clostridium difficile

A previously described protocol was used for isolation of* C. difficile* on cycloserine-cefoxitin fructose agar supplemented with 7% horse blood and 0.1% sodium taurocholate (Sigma, USA) [24]. After anaerobic incubation for 72 h at 37°C, all colonies with characteristic* C. difficile* morphology (flat, irregular, and with ground-glass appearance) were subjected to multiplex-PCR for detection of housekeeping gene (*tpi*), toxin A gene (*tcdA*), toxin B gene (*tcdB*), and binary toxin gene (*cdtB*) (Table 1S of Supplementary Material). The reference strain* C. difficile *ATCC 9689 were used as control.* C. difficile* strains were submitted to PCR ribotyping, a molecular tool largely used for* C. difficile *typing since 1990s [25]. Intergenic spacer regions were amplified using Bidet primers, as previously described [26]. Amplification products were separated by electrophoresis in a 3% agarose gel (Bio-Rad, USA) for 5 h at 2.5 V/cm, and the gel was photo-documented and then analyzed using BioNumerics 7.6 (Applied Maths, Belgium). The PCR ribotypes were designated by the international Cardiff nomenclature. Multilocus sequence typing (MLST) was conducted as previously described by Griffiths et al. [27], using a typing scheme with discriminatory power similar to PCR ribotyping [25], being the following housekeeping genes:* adk, atpA, dxr, glyA*,* recA, sodA*, and* tpi*. Amplicon sequences were compared with the MLST database (https://pubmlst.org/cdifficile/) to identify the allelic profiles and the corresponding Sts. The minimal inhibitory concentration (MIC) for metronidazole, vancomycin, clindamycin, and sulfamethoxazole/trimethoprim, commonly associated with CDI was performed by gradient test with the M.I.C. Evaluator™ (M.I.C.E.™) strips (Oxoid, USA). Briefly, a suspension of the toxigenic strains of* C. difficile* was prepared on sterile 0.9% saline to McFarland standard 1 from a pure culture after 24 hours' growth in Brucella agar (Oxoid, USA). The test was performed on Brucella agar with 5% lysed blood supplemented with hemin (Difco, USA) and vitamin K (Sigma, USA). The plates were incubated at 37°C in anaerobic atmosphere and the MIC end point were measured and interpreted according to cut-off values from M100-S25 [28] and EUCAST guidelines [29] after 48 h of incubation.

### 2.6. Statistical Analysis

Associations between the categorical variables (order or suborder of reptile, diet, and habitat of reptile) and the frequency of microorganisms identified were studied using univariate analysis with the Chi-square test or Fisher's exact test within Stata 12® software (StataCorp, USA), with a* p* value of ≤0.05 being considered significant.

## 3. Results

*E. coli* was isolated from 52 (68.4%) out of 76 sampled animals, of which 38 (50%) isolates were from snakes, 7 (9.2%) were from lizards, 4 (5.2%) from the suborder Pleurodira, and 3 (3.9%) from the Cryptodira. When compared to lizards (*p *= 0.003), suborder Pleurodira (*p *= 0.002) and Cryptodira (*p *= 0.04), the frequency of isolation of* E. coli *was higher in snakes (Table 1). Of the free-living and pet reptiles, 62.1% and 65.2% were positive for* E. coli, *respectively. The frequency of isolation in captivity reptiles was 87.5%, and no statistic difference was found among the positivity of* E. coli *according to the reptile habitats (Table 2). The isolation frequency of* E. coli* in carnivores was significantly higher than for omnivorous and herbivorous reptiles (*p* = 0.01) (Table 2). Considering phylogenetic groups, almost 89% (46/52) of the* E. coli* strains belonged to the B1 group (Table 1). It was found a greater propensity of isolation of B1* E. coli* from snakes and lizards (*p* = 0.00004) while the groups A and B2 were more frequent in chelonians (*p* = 0.0001). Also, there is a positive association between phylogroup B1 and* E. coli *strains isolated from carnivorous reptiles (*p = *0.0001). Of the sampled reptiles, approximately 9.2% were positive for the virulence gene* cnf1 *of NTEC pathovar, corresponding to seven positive strains (13.4%) from B1, B2, and F groups. The virulence factor gene encoding EAST1 (enteroaggregative* E. coli* heat-stable enterotoxin 1) was identified in two (3.8%) strains, classified as F and B1, respectively (Table 3). Three positive strains for CNF1 were resistant to cephalothin, one shows intermediate resistance to cephalothin and the last one strain, isolated from a wildlife testudine, was resistant to cephalothin, ciprofloxacin, trimethoprim/sulfamethoxazole, and chloramphenicol. On the other hand, the EAST1 strains were sensitive to all antimicrobial agents tested. Two* E. coli cnf1+* strains were isolated from a lizard and a snake that were also positive for* S. *Johannesburg and* S*. Ndolo, respectively. Additionally, one toxigenic* C. difficile *strain was isolated from a pet testudine that carried* E. coli cnf1+* with intermediate resistance to cephalothin. One reptile was simultaneously positive for* E. coli, S. enterica houtenae, *and nontoxigenic* C. difficile, *while other two were positive for* S. enterica *subsp* enterica, E. coli, *and* C. perfringens *type A.

*Salmonella *spp. was isolated from 26 (34.2%) reptiles, with higher rates of carriage in lizards and snakes than chelonians (*p* = 0.007) (Table 4), and in carnivores (*p* = 0.003) and herbivores (*p* = 0.003) than omnivores (Table 2). Of the captivity reptiles 50% were positive for* Salmonella *spp., while the frequency of isolation in companion animals and free-living reptiles were respectively 30.4% and 29.7% (Table 2) and no statistic difference was found between the different habitats. All 26* Salmonella* isolates were classified as* S. enterica* with 13 strains belonging to the subspecies* enterica* (Table 4). The other* S. enterica* isolates comprised the subspecies* houtenae*,* arizonae*, and* diarizonae*. Ten different serovars of* S. enterica* subspecies were identified in the present study, including the important zoonotic serovars* S*. Infantis,* S. *Mbandaka,* S*. Heidelberg, and* S*. Panama (Table 5). All but one of the* Salmonella *isolates were sensitive to the four classes of antimicrobial agents tested. The exception was a strain of* S. enterica *subsp.* houtenae*, obtained from a domesticated lizard (*Iguana iguana*), which was resistant to cephalothin and ciprofloxacin. This domiciled lizard was also positive for EAST1* E. coli*.

*C. perfringens* was isolated from six animals (7.8%) and no difference in isolation between captivity, free-living, or pet reptiles was identified (Table 3). The alpha toxin gene (*cpa*) was identified in each of these strains, being classified as* C. perfringens *type A. Not additional virulence factors tested were identified.

*C. difficile* was isolated from three (3.9%) reptiles, being two toxigenic strains (A+B+CDT-) and one nontoxigenic (A-B-CDT-) (Table 3). One of the toxigenic strains, identified as ribotype (RT) 081 and strain type (ST) 09, was recovered from a captive snake (*Bothrops alternatus*). The other toxigenic strain was isolated from a domesticated chelonian from the suborder Pleurodira (*Phrynops geoffroanus*) and classified as RT106 and ST042. The two toxigenic strains were susceptible to metronidazole, vancomycin and sulfamethoxazole/trimethoprim, but resistant to clindamycin. The nontoxigenic* C. difficile* strain was isolated from a pet corn snake (*Pantherophis guttatus*) and was classified as RT009 and ST457.

## 4. Discussion

Being a common inhabitant of the intestinal tract of warm-blooded vertebrates,* E. coli *can also be isolated in cold-blooded animals, such as reptiles, which frequency is highly dependent on their diet and contact with other animals [8]. The frequency of isolation of* E. coli *found in the present study was higher than reported in reptiles by previous studies [8, 9] although these reports have stated different methodologies and objectives. The higher frequency in snakes when compared to other reptiles might be due to the diet of these animals, since all snakes are carnivores. Interestingly, these results contrast previous studies that indicate that* E. coli *is more likely to be isolated from omnivorous mammals [8]; however, the present results could be influenced by the low number of omnivores in the sample population.

Each of the* E. coli* isolates from reptiles was classified into one of the seven phylogroups (A, B1, B2, C, D, E, and F) according to the quadruplex PCR developed by Clermont et al. [16]. The high frequency of* E. coli *isolates belonging to group B1 was similar to the only previous study evaluating phylogroups of* E. coli *isolates from reptiles [8]. This finding also corroborated previous reports that show that most* E. coli* from animals belong to group B1, whereas, in humans, groups A and B2 are predominant [10]. Previous studies have already suggested that phylogenetic groups are associated with different hosts [30], which could justify the greater propensity for isolation of B1* E. coli *from snakes and lizards, already suggested by Gordon and Cowling [8], as well as the higher frequency of groups A and B2, commonly described in humans, from chelonians. Curiously, all samples that were identified as A or B2 strains were isolated from pet reptiles, raising the question of whether close contact of reptiles and humans may have resulted in colonization of these animals with phylogroups commonly associated with humans.

In mammals,* E. coli *genotype distribution seems to depend on several factors, including the climate, host diet, and host body mass. In addition, phylogroup B1 is more common in carnivorous mammals, probably due to the lower complexity of their gastrointestinal tract [8, 30]. Although the present study analyzed isolates from carnivorous reptiles, the results suggested that, as demonstrated for mammals, the diet may also influence in the phylogenetic distribution of* E. coli *isolated from these animals.

Pathogenic strains of* E. coli *have been reported as the causative agent of intestinal and extra intestinal diseases in humans and animals, although there have been no studies focusing on the detection of pathogenic* E. coli *in reptiles. Therefore, to the best of our knowledge, this is the first report of the CNF1 and EAST1 factors in reptilian* E. coli* strains.* E. coli cnf1*^+^ are responsible for diarrhea and extra intestinal diseases such as cystitis and meningitis in humans and domestic animals [31–33], while EAST1 positive strains have been associated with several outbreaks of diarrhea in humans [34]. Thus, the present study suggests that reptiles positive for EAST1 and CNF1 strains may represent a risk for human and animals health. As described, five* cnf1+* strains were not susceptible to cephalothin, an antimicrobial agent that could be used for treatment of urinary tract infections (UTI) in humans [35, 36]. Considering their resistance to cephalothin, ciprofloxacin, trimethoprim/sulfamethoxazole, and chloramphenicol, the* cnf1+ *strain isolated from a testudine was classified as multidrug resistant (MDR) [37]. The occurrence of MDR strains is of high public health concern, since it could contribute to therapeutic failure and increased patient morbidity and mortality [38]. Curiously, ciprofloxacin is a fluoroquinolone recommended for complicated infections and urinary tract infections (UTI) caused by MDR Gram-negative bacteria, such as* E. coli *[36]. Additionally, chloramphenicol and trimethoprim/sulfamethoxazole are also common choices for treatment of UTI and diarrhea associated with* E. coli *in humans and animals [36, 39, 40].

*Salmonella enterica* has been described as an animal and human enteropathogen. There are six known subspecies of* S. enterica *(*enterica*,* salamae, arizonae, diarizonae, houtenae*, and* indica*) and more than 2500 serovars that have been associated with different types of infection [20]. Previous reports in other countries, including a single study in Brazil, have showed that* Salmonella* shedding by reptiles is frequent, suggesting that they are natural hosts that eventually become ill [41–43]. It is important to highlight that only one cloacal swab was collected from each animal in the present work. Since it is known that* Salmonella* shedding by reptiles is intermittent [44], the number of asymptomatic colonized reptiles might be much higher than the 34% reported here thus may represent some risk to the carrier as well as to people in close contact with these animals [2].

Previous reports have shown a highest shedding of* Salmonella *spp. by carnivores' reptiles, a result also found in the present study when compared to omnivorous reptiles [45]. This result was also found for* E. coli *shedding, which may have influenced the higher rate of cocolonization of these animals in contrast with omnivore and herbivore reptiles, most of them from Chelonii order. Curiously, previous reports have shown that shedding of* Salmonella *spp. is greater in carnivore reptiles fed by contaminated reptile feeder mice [46]. In fact, feeder rodents are potential carriers of* Salmonella *spp. [47]. On the other hand, the higher carriage rate of* Salmonella *spp. by herbivores compared to omnivores may not represent what actually occurs in nature, considering the small sampling of herbivores reptiles in the present study (five samples, 6.5% of the total), of which three were positive for* Salmonella *spp.

The higher* Salmonella *carriage rate among lizards and snakes compared with chelonians is consistent with findings of previous studies [41]. Chelonians have been recognized as sources of human salmonellosis since the mid-1960s, mainly due to their popularity as pets [48]. On the other hand, special attention has been recently given to lizards and snakes, since the domestication of these reptiles has considerably increased [49], even in spite of the indication by several studies that these animals seem to have a high rate of* Salmonella *shedding [4, 42, 50]. This concern seems legitimate, since a recent report has shown that lizards became an important source of* Salmonella *spp. in human reptile-associated salmonellosis [51].

The subspecies* houtenae*,* arizonae*, and* diarizonae*, which corresponded to 50% of the isolates of* S. enterica *identified in the present study, are common in cold-blooded animals [4] and are occasionally associated with human salmonellosis [52, 53]. On the other hand,* Salmonella enterica *subsp.* enterica* is the most common subspecies associated with human disease [45]. Notably, half of the* Salmonella* strains isolated from reptiles in the present study belonged to the subspecies* enterica,* a proportion higher than described in other studies [43, 54]. This difference might be due to several factors, including the geographical origin of the animals, sampling, and living conditions (captive, as pets, or free-living) that these reptiles were subjected to (Table 2) [41, 45].

Several serotypes of* Salmonella* are recognized as etiological agents of reptile-associated salmonellosis in humans [43, 48, 51, 53]. In fact, half of the serotypes identified here have been reported to infect humans (Table 5). Some of them (including* S. *Mbandaka,* S. *Panama, and* S*. Infantis) are listed as the most common isolates associated with human infection in Brazil [55, 56], and in the United States and European countries, including* S. *Heidelberg,* S. *Panama, and* S*. Infantis [57, 58]. It should also be highlighted that almost 70% of the serotypes identified in the present study, including* S*. Heidelberg,* S*. Infantis, and* S*. Mbandaka, were isolated from pet reptiles that were kept in close contact with humans. Also, this study seems to be the first to report isolation of* S*. Johannesburg and* S*. Ndolo from reptiles, serotypes already described to infect humans in Brazil [56, 59].

Resistance to cephalothin and ciprofloxacin, found in one strain of* S*.* enterica* subsp.* houtenae, *may make it difficult to treat salmonellosis in humans [28]. As described, ciprofloxacin is a fluoroquinolone critically important in human medicine and largely used to treat serious infections. Additionally,* Salmonella* fluoroquinolone-resistant is one of 12 bacterial agents for which new antibiotics are urgently needed [60]. It is important to note that this resistant strain of* Salmonella *was isolated from a pet reptile also positive for EAST1* E. coli, *thus reinforcing the possible risk of these animals for human health. Altogether, our results demonstrate the importance of reptiles as reservoirs of* Salmonella *spp. and* E. coli *and highlight the need to study these agents in view of One Health.

*C. perfringens* is a widespread gram-positive anaerobic bacillus, commonly found as part of the microbiota of animals and humans [61]. However, there is little information regarding the occurrence of* C. perfringens *in reptiles. Most studies are restricted to its isolation from the oral microbiota and venom of some snake species [62, 63] or on the effect of diet on the occurrence of clostridia species [64]. In the present study, the frequency of reptiles positive for* C. perfringens* was much lower than previously reported for other animal species, which is commonly above 75% [65, 66]. These results suggest that* C. perfringens* is less frequently isolated from the microbiota of reptiles than from that of most warm-blooded animals.

*C. perfringens *may also be classified into five types (A to E) according to the production of four major toxins: alpha, beta, epsilon, and iota [23]. In addition to the major toxins, the bacterium produces additional virulence factors that are associated with the pathogenesis of some diseases in humans and animals, such as enterotoxin, beta-2 toxin, NetB, NetE, NetF, and NetG [67, 68]. Interestingly, no additional virulence factors tested were identified, including the enterotoxin-encoding gene (*cpe*), which is commonly associated with disease in humans [61] and is already suggested as a cause of diarrhea in red-footed tortoise (*Geochelone carbonaria*) [13]. Thus, considering the small frequency of isolation of* C. perfringens *and the absence of additional virulence factors in these isolates beyond the alpha toxin encoding gene, present in all* C. perfringens *strains, the present work suggests that the fecal shedding of this agent by reptiles may not represent a public health concern.

*C. difficile* is an anaerobic gram-positive bacterium considered an emerging pathogen, being responsible for the majority of nosocomial diarrhea cases in humans [11]. In animals,* C. difficile* infection (CDI) is common in piglets and horses, but also occurs in several other species, including some wild animals [12, 69]. There are few studies on* C. difficile* shedding by wild animals and, to the best of our knowledge, this is the first report on toxigenic* C. difficile* isolated from reptilian species. Some other free-living or captive species are reported as possible reservoirs of* C. difficile* strains, but the role of these animals in the epidemiology of CDI in humans and domestic species, in addition to the risk for reptiles' health, is still poorly understood [12, 65, 70, 71]. It is important to note that none of the positive animals were undergoing antibiotic therapy which is known to increasing the shedding of* C. difficile* in humans and animals [11, 69], including wild species [65].

The nontoxigenic* C. difficile* strain found in the present study, classified as RT009, was described with a high capacity to colonize different host species, being one of the most common ribotypes isolated from humans and domestic animals [72]. Interestingly, this isolate was classified as ST457, a novel strain type of* C. difficile*. The only study of* C. difficile *in reptiles was recently published, describing another nontoxigenic* C. difficile* strain isolated from a lizard (*Pogona vitticeps*) that belonged to a new ribotype and to strain type 347 [12]. The two toxigenic RTs and STs identified in the present study have been described in strains isolated from humans with CDI worldwide, including Brazil [72, 73]. Of note, several studies have demonstrated high similarity between* C. difficile* isolates obtained from humans and companion animals, suggesting a possible zoonotic transmission [11, 69, 74].

Regarding the antimicrobial susceptibility of the toxigenic* C. difficile* isolates, both strains were sensitive to metronidazole and vancomycin, the most common antimicrobials used to treat CDI in humans and some animals' species [75]. On the other hand, both strains were resistant to clindamycin. This is not surprisingly once clindamycin has been linked to* C. difficile *infection due to microbiota changes but also to the common resistance of* C. difficile* strains to this antimicrobial [75, 76].

## 5. Conclusion

The present study demonstrates the potential of reptiles in Brazil to carry human and animal enteropathogens other than* Salmonella *spp., including toxigenic* C. difficile* and potentially pathogenic* E. coli*. Further studies with probabilistic sampling of reptiles are necessary to better elucidate the true prevalence of these enteropathogens in reptiles from Brazil, thus clarifying the role of reptiles as reservoirs of enteropathogens.

## Figures and Tables

**Table 1:** Frequency of isolation and phylogenetic groups of *E. coli* from reptiles in Brazil.

Host group	No. (%) *E. coli*	No.* E. coli* groups*∗* (%)				
		A	B1	B2	D	F
Serpentes (n=45)	38/45 (84.4)	0	38/38 (100)	0	0	0

Sauria (n=15)	7/15 (46.6)	1/7 (14.2)	5/7 (71.4)	0	0	1/7 (14.2)

Cryptodira (n=6)	3/6 (50)	1/3 (33.3)	2/3 (66.6)	0	0	0

Pleurodira (n=10)	4/10 (40)	0	1/4 (25)	2/4 (50)	1/4 (25)	0

Total	52/76 (68.4)	2/52 (3.8)	46/52 (88.4)	2/52 (3.8)	1/52 (1.9)	1/52 (1.9)

*∗* None *E. coli* strains belonged to phylogenetic groups C and E.

**Table 2:** Frequency of isolation of *E. coli* and *Salmonella* spp. from reptiles based on dietary habits and animal habitats.

Dietary habit	No. Samples	No.* E. coli-*positive (%)	No.* Salmonella*-positive (%)
Carnivore	52	40/52 (76.9)	22/52 (42.3)
Herbivore	5	4/5 (80)	3/5 (60)
Omnivore	19	8/19 (42.1)	1/19 (5.2)

Animal habitat	No. Samples	No.* E. coli-*positive(%)	No. *Salmonella*-positive (%)

Pet	23	15/23 (65.2)	7/23 (30.4)
Captivity	16	14/16 (87.5)	8/16 (50)

Wild	37	23/37 (62.1)	11/37 (29.7)

**Table 3:** Frequency and characteristics of *E. coli*, *Salmonella* spp., *C. difficile* and *C. perfringens* isolated from reptiles in Brazil.

Host group	*E. coli (%)*			*Salmonella spp. (%)*	*C. difficile (%)*		*C. perfringens Type A*
	Isolates	EAST1	CNF1		Isolates	A+B+	
Sauria (n=15)	7 (46.6)	2 (28.5)	3 (42.8)	7 (46.6)	0	0	2 (13.3)

Serpentes (n=45)	38 (84.4)	0	1 (2.6)	18 (40)	2 (4.4)	1 (50)	3 (6.6)

Pleurodira (n=10)	4 (40)	0	3 (75)	1 (10)	1 (10)	1 (100)	1 (10)

Cryptodira (n=6)	3 (50)	0	0	0	0	0	0

Total (n=76)	52 (68.4)	2 (3.8)	7 (13.4)	26 (34.2)	3 (3.9)	2 (66.6)	6 (7.8)

**Table 4:** Frequency of isolation of *Salmonella enterica* subspecies from reptiles in Brazil.

Host group	No. samples	No (%) *Salmonella*	No (%) *Salmonella enterica *subspecies			
			*enterica*	*houtenae*	*arizonae*	*diarizonae*
Sauria	15	7/15 (46.6)	3/7 (42.8)	4/7 (57.1)	0	0
Serpentes	45	18/45 (40)	10/18 (55.5)	4/18 (22.2)	2/18 (11.1)	2/18 (11.1)
Pleurodira	10	1/10 (10)	0	1/1 (100)	0	0
Cryptodira	6	0	0	0	0	0

Total	76	26/76 (34.2)	13/26 (50)	9/26 (34.6)	2/26 (7.6)	2/26 (7.6)

**Table 5:** * Salmonella enterica* serotypes isolated from reptiles.

Serotype	No. of isolates	Host	Origin
Anatum	1	Serpentes	Captivity
Heidelberg	1	Serpentes	Captivity
Infantis	1	Serpentes	Captivity
Johannesburg	1	Sauria	Wild
Mbandaka	2	Serpentes and Sauria	Pet and Captivity
Ndolo	3	Serpentes	Captivity
Panama	1	Serpentes	Wild
16: - : -	1	Serpentes	Wild
6,7: - : -	1	Sauria	Pet

Rough: - : -	1	Serpentes	Wild

## Data Availability

The results and statistical analysis that support the findings of this study are included within the article.

## Supplementary Materials

Supplementary Materials The primers used to detect genes encoding virulence factors associated with diarrheagenic* E. coli *in humans and animals and their references are described in Table S1 in the Supplementary Material.
